# Overweight/Obesity in Childhood and the Risk of Early Puberty: A Systematic Review and Meta-Analysis

**DOI:** 10.3389/fped.2022.795596

**Published:** 2022-06-03

**Authors:** Xuan Zhou, Yang Hu, Ziqi Yang, Ziqiang Gong, Senmao Zhang, Xiaoling Liu, Yan Chen, Changxiang Ye, Lizhang Chen, Tingting Wang

**Affiliations:** ^1^Department of Epidemiology and Health Statistics, Xiangya School of Public Health, Central South University, Changsha, China; ^2^Department of Plastic Surgery of Third Xiangya Hospital, Central South University, Changsha, China; ^3^Hunan Provincial Key Laboratory of Clinical Epidemiology, Changsha, China; ^4^NHC Key Laboratory for Birth Defect for Research and Prevention, Hunan Provincial Maternal and Child Health Care Hospital Hunan, Changsha, China

**Keywords:** meta-analysis, childhood, early puberty, puberty time, overweight, obesity

## Abstract

**Purpose:**

To determine the relationship between childhood overweight/obesity and early puberty in both boys and girls. Specifically, this is the first time to conduct a meta-analysis of the relationship between childhood overweight/obesity and early puberty in boys.

**Methods:**

Relevant studies were identified from PubMed, Web of Science, and EMBASE searches. The exposure of interest was overweight/obesity in childhood. Childhood was defined internationally as the age range of 0–18 years. The overall risk estimates were pooled using random effects models. Subgroup and sensitivity analyses were performed to explore possible sources of heterogeneity and to assess the robustness of the results.

**Results:**

A total of 10 studies involving 13,338 girls and 12,796 boys were included. Results showed that childhood overweight/obesity were associated with a significantly higher risk of early puberty in girls [odds ratio (OR): 2.22, 95% CI: 1.65–2.99]. Although without statistical significance, a higher risk of early puberty was also found in boys who were overweight/obese in childhood (OR: 1.29, 95% CI: 0.98–1.70). Heterogeneity in the risk estimates of early puberty was partially explained by study design, sample size, follow-up duration, definitions of early puberty and confounders controlled. Sensitivity analyses validated the robustness of the findings.

**Conclusions:**

Our findings showed that for girls the associate between overweight/obesity and early puberty is definite or strong whereas for males, such an association is possible, prompting that future studies need to further explore the possible relationship between overweight/obesity and early puberty in boys.

**Systematic Review Registration:**

https://www.crd.york.ac.uk/prospero/display_record.php?ID=CRD42021264649, PROSPERO CRD42021264649.

## Introduction

Overweight/obesity in childhood is a global public health problem that is prevalent in both developing and developed countries (1, 2). As reported, up to 19.2% individuals aged 7–18 years old were overweight or obese in China (3). In the United States, the prevalence of obesity in 6 to 11-year-old children rose from 7% in 1980 to nearly 18% in 2012. Concurrently, the percentage of obese individuals aged in the 12 to 19-year-old population rose from 5 to nearly 21% (4). In 2016, more than 340 million individuals aged 5–19 were overweight or obese worldwide (5).

Childhood overweight/obesity is widely regarded as a risk factor for comorbidities in childhood or later in life, such as type two diabetes, hypertension, dyslipidemia, coronary heart disease, hepatic and orthopedic problems, and psychosocial comorbidities (6). In addition, studies have shown that childhood overweight/obesity may also have adverse effect on the process of puberty (7–10). However, the findings were discrepant. In a longitudinal study from the United States, girls who had a higher body mass index (BMI) at 36 months were more likely to exhibit an earlier puberty relative to girls of normal weight (11). Similar results were found among boys. The Swedish Young Male Twins Study showed that boys who matured early had a higher BMI through childhood (12). A European study also reported a positive relationship between preadolescent BMI and early puberty in boys, where voice breaking was defined as puberty occurring (13). Nevertheless, in the study performed in American boys, there was a negative association between childhood obesity and early puberty (14). Additionally, several studies did not find a significant association between childhood obesity and early puberty (15–17). Considering the adverse effects of early puberty on later reproductive and physical health, including lower fertility as well as higher risks of type two diabetes, cardiovascular disease, and cancer, it is necessary to determine the relationship between childhood overweight/obesity and early puberty in both boys and girls.

A previous meta-analysis published in 2017, including five cohort studies with a total of 1,360 girls, has showed that early puberty was significantly associated with childhood overweight/obesity (RR = 2.44) (18). After the publication of this study, two cohort studies with a large sample size (3,109 and 1,126 individuals respective) were performed (19, 20). Both of them showed relatively lower risk estimates for early puberty than the estimate in the published meta-analysis, with an odds ratio of 1.11 and 1.79, respective. Therefore, updating the extent of the association of childhood overweight/obesity with risk of early puberty in girls is necessary, which would help to add important data to the knowledge base. In addition, there is currently no study to quantitatively summarize the associations between childhood overweight/obesity and early puberty in boys; this lack of information is a major obstacle for the risk assessment of early puberty in children who are assessed as overweight/obesity.

Therefore, the objective of this study was to perform a comprehensive meta-analysis regarding the association between childhood overweight/obesity and risk of early puberty in both girls and boys. The result of our study may lead to a better understanding of early puberty risk in children who are overweight or obese.

## Materials and Methods

### Search Strategy

We conducted the present meta-analysis according to the Preferred Reporting Items for Systematic Reviews and Meta-Analysis (PRISMA) Statement (21). Two authors independently screened studies published in English prior to 10 January 2021, which reported overweight/obesity and early puberty. PubMed, Web of Science, and EMBASE were systematically searched. The following search terms were applied in combination: (1) weight, height, body mass index, and BMI; (2) infant, infancy, early, postnatal, childhood, preschool, pre-school, and school; (3) overweight, obesity, adiposity, body composition, and waist circumference; and (4) puberty, adrenarche, pubescence, menarcheal, adolescence, and youth. Reference lists of all reviews related to the topic and other included studies were manually searched for additional probably eligible studies.

### Inclusion and Exclusion Criteria

In our study, the key exposure variable was overweight/obesity in childhood, and the key outcome variable was early puberty. Childhood was defined internationally as the age range of 0–18 years. The inclusion of titles and abstracts was intentionally expanded with a view to obtaining more relevant studies. First, studies would be taken into account if they reported on overweight/obesity and early puberty and were published in English. Then, full texts of the selected studies were reviewed. Studies were included if they: (1) were observational in design; (2) had clear definitions of exposure and outcome; (3) reported on the association between overweight/obesity in childhood and early puberty; and **(**4) had sufficient information to calculate risk estimation and 95% confident intervals (CIs). Studies were excluded if they: (1) were reviews, case reports, letter to the editor, or abstracts; (2) provided vague or incomplete data; (3) were redundant publications; or (4) included children with any diseases which would affect pubertal development as participants. If there was more than one study from the same population, only the most comprehensive or most recent published one was included.

### Data Abstraction

Two reviewers independently extracted and evaluated the data for each included article using a self-designed data abstraction form. Any disagreements between the two reviewers will be resolved through discussion or consultation with a third reviewer. The following data were extracted: first author, year of publication, study period, geographic region, gender, study design, length of follow-up, and age of enrolment (for cohort studies only), sample size, definition of overweight/obesity, definition of early puberty, and adjustments and matches made. The most important difference between cohort studies and case-control studies is the ability to demonstrate causation. Cohort studies group subjects according to whether they are exposed to a particular study factor and observe whether a specific outcome occurs in each group after longitudinal observation. This sequence conforms to the law of natural development of causality, so it has a strong ability to test the etiology hypothesis. However, case-control studies divided subjects into groups according to whether they suffered from a specific disease or not, and analyzed the association between exposure and outcome through retrospective collection of exposure to a specific factor in the past in each group. It was often difficult to determine the time sequence of exposure and outcome, so its causal verification ability was weak. Secondly, case-control studies are prone to recall bias. For example, when case-control studies are used to explore the relationship between smoking and lung cancer, lung cancer patients are likely to have recall bias on their past smoking dose and frequency due to the long incubation period of lung cancer.

### Quality Assessment

The methodological quality of studies was assessed using the Newcastle-Ottawa Scale (NOS) for quality assessment of cohort and case-control studies by two researchers (XZ and YH.) independently. The NOS was composed of eight items. It ranged from 1–9 stars and assessed the quality of each study based on three modules: selection, comparability, and outcome (cohort studies) or exposure (case-control studies). A final median score of ≥ 6 was regarded as high quality.

### Statistical Analysis

Odds ratio (OR) was used as a reference value to measure the association between overweight/obesity and early puberty. Random effects model meta-analysis was used as a tool to calculate combined ORs and 95% CIs (22). Heterogeneity was assessed by the Chi-square test (*p* < 0.05 represented statistically significant heterogeneity) and *I*^2^ statistic (*I*^2^ > 75% indicated an extremely high level of heterogeneity, 51–75% showed a high level of heterogeneity, 26–50% demonstrated a moderate-level of heterogeneity, and ≤ 25% signified a low level of heterogeneity) (23, 24). The Chi-square test was used to estimate whether the variance between studies was ascribed to chance and the *I*^2^ statistic was used to estimate the proportion of total variation in prevalence evaluations owing to statistical heterogeneity instead of sampling error. Publication bias of included articles was assessed by Begg's test (*p* < 0.05 indicated statistically significant differences). Separate subgroup analyses were performed on the different categories to detect possible sources of heterogeneity, including geographic region (e.g., Europe, Asian, North America), study design (e.g., cohort, case-control), sample size (e.g., > 2000, ≤ 2000), follow-up duration (e.g., > 8 years, ≤ 8 years), age at enrolment (e.g., ≥ 7 years old, **<** 7 years old), type of exposure (e.g., obesity, overweight), definition of overweight/obesity (e.g., body mass index, skinfold thickness, body fat), definition of early puberty [e.g., age of menarche, age of breast stage II stage, three criteria in the study, age of each Tanner stage, puberty development scale (PDS) score > $\bar{x}$ + s ], and confounders controlled (e.g., none of the confounder controlled, one or more confounders controlled). Sensitivity analyses were conducted to evaluate the robustness of the meta-analysis results, i.e., repeating meta-analyses were conducted after excluding one included study at a time and comparing the results before and after exclusion. All the statistical analyses were conducted by RevMan version 5.4.1 (The Nordic Cochrane Center, Cochrane Collaboration, Copenhagen, Denmark) and R version 4.0.5 (The R Foundation for Statistical Computing).

## Results

### Selection and Characteristics of the Studies

**Figure 1:**
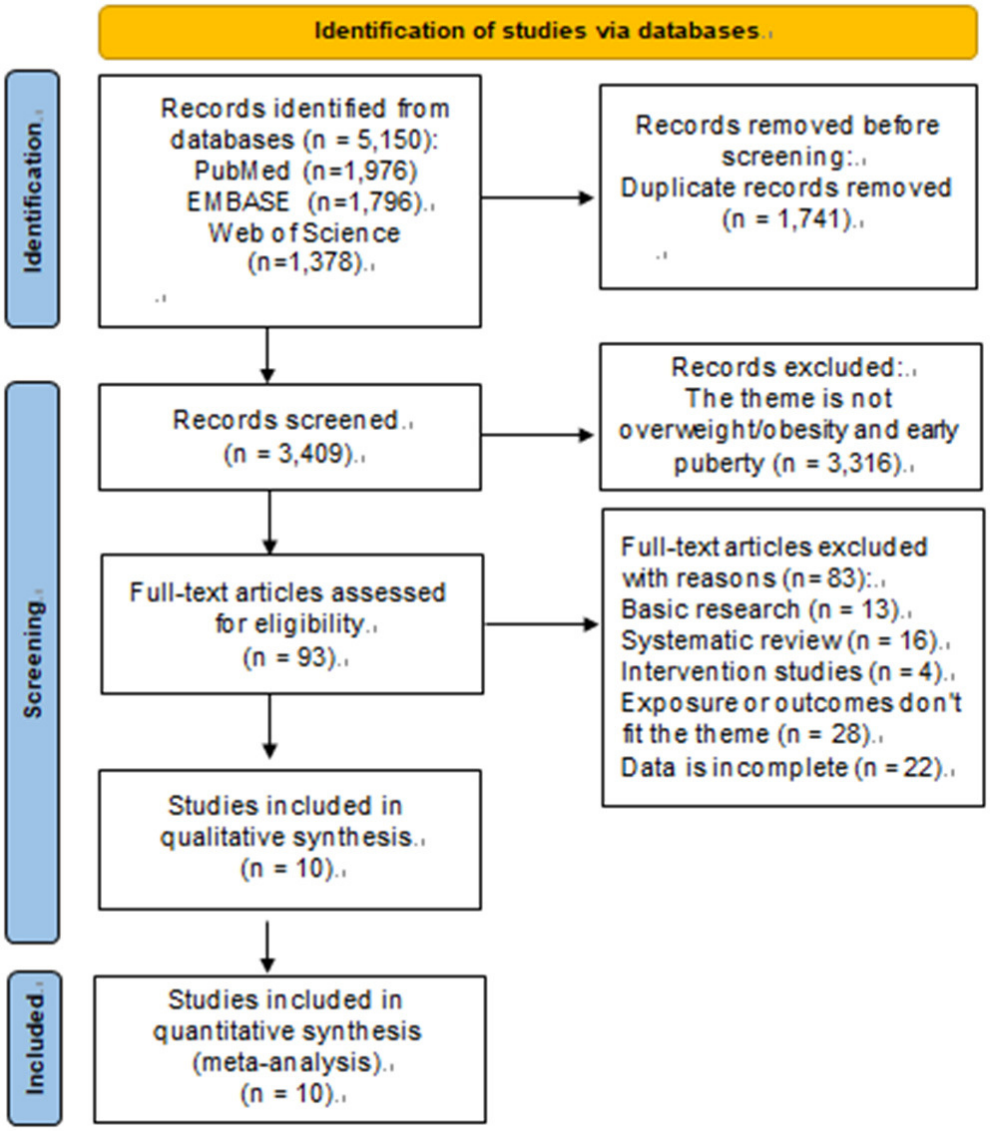
PRISMA flow diagram.

A total of 5,150 articles were retrieved through the system search and manual search. Of these, 3,409 articles were left after eliminating the duplicate publication, 3,316 articles were excluded after screening the title and abstracts, and 83 of them were excluded after screening the full text. Finally, 10 articles were included in meta-analysis (see Figure 1) (13, 19, 20, 25–31).

The characteristics of the 10 included studies are shown in the (Tables 1, 2). Included studies were published between 2002 and 2019, involving a total of 13,338 girls and 12,796 boys. Of the observational studies included here, seven were cohort in design and three were case-control. Among the studies, five were conducted in Asia, four in North America, and one was conducted in Europe. For these cohort studies, the group age of subjects ranged from 4 months to 11 years, and the follow-up time ranged between 3 and 18 years. Most studies used BMI as the basis for defining childhood overweight/obesity, a few studies used body fat and skinfold thickness. Almost all studies defined early puberty by menarche age, while Chen et al. (27) used Tanner stage of breast or public hair. Among all studies included, four studies evaluated the association of childhood overweigh/obesity with early puberty in both boys and girls (19, 27–29), five studies only evaluated that in girls (20, 25, 26, 30, 31), and one study only evaluated that in boys (13). A number of studies controlled for one or more confounding factors (13, 19, 20, 25, 26, 29, 31), such as parental education, race, height, whether the only child, family income, birth weight, breastfeeding, gestational age, and in-utero smoking. All studies included here were of medium or high quality. The median quality score of the included studies was eight.

**Table 1:** Selected characteristic of 7 cohort studies of childhood overweight/obesity and early puberty.

**References**	**Study period**	**Geographic region**	**Gender**	**Age of enrolment (years)**	**Follow-up (years)**	**Sample size**	**Definition of overweight/obesity**	**Definition of early puberty**	**Adjustments or matches made**	**Quality score**
Chen et al. (19)	2010–2018	China	Girls and boys	10	8	3,109	Obesity: BMI of ≥95 percentile Overweight: 85th BMI to 94th percentile	Early menarche was defined as age at menarche earlier than 12 years. In addition, early voice breaking was defined as age at voice breaking earlier than 13 years	Parental education, family income, birth weight, breastfeeding, gestational age, and in-utero smoking.	9
Davison et al. (26)	1996–2000	America	Girls	5	4	181	Obesity: According to 2000 growth charts from the Centers for Disease Control and Prevention	Earlier developers were girls who fulfilled at least two of the following three criteria: (1) highest tertile for estradiol, (2) tanner stage three for breast development; and (3) highest tertile for the PDS	Height	7
Flom et al. (20)	1967–1985	America	Girls	4 months	18	1,126	Obesity: CDC cut-point for the 85th percentile of BMI (17.626 kg/m2)	Menarche year <12	Maternal age at pregnancy, menarche, pre-pregnancy weight and BMI, height, pregnancy weight gain, prenatal tobacco smoke exposure, education, race/ethnicity and birth size	9
Lee et al. (13)	1991–2003	America	Boys	2	11.5	401	Obesity:BMI ≥95th percentile Overweight: >85th BMI <95th percentile at age 11.5	Tanner two development by ages 9.5 and 10.5 years	Total family income, race	8
Leitão et al. (31)	1998–2006	Portugal	Girls	7	8	109	Obesity: The health-related criterion (≥ 30 % body fat)	Age at menarche cut-offs were <12 years	Urban/rural ratio, socio-economic status	9
Tremblay and Frigon (30)	1986–1997	Canada	Girls	11	3	811	Obesity: BMI ≥ 95th percentile Overweight: Children's BMI between 86th and 95th percentile	Menarche years <11	None	7
Zhai et al. (25)	1999–2003	China	Girls	7	4	80	Obesity: The percentage of body fat using skinfold thickness: ≥25%	Breast stage II stage earlier than the median age(9.2 years)for that stage in China	Matched by height, school grade, Tanner stage, family economic status	8

**Table 2:** Selected characteristic of 3 case-control studies of childhood overweight/obesity and early puberty.

**References**	**Geographic region**	**Gender**	**Sample size**	**Definition of overweight/obesity**	**Definition of early puberty**	**Adjustments or matches made**	**Quality score**
Chen et al. (27)	China	Girls and boys	15,937	Obesity: WHO Child Growth Standards BMI ≥2 *SD* Overweight: WHO Child Growth Standards BMI ≥1 *SD*	Under 8 years for Tanner stage two or above for breast (B2) or pubic hair development (PH2), 10 years for menstruation in girls, and under 9 years for Tanner stage two or above for pubic hair or testicle development (T2) (testicular volume, TV ≥4 mL) in boys	None	8
He et al. (28)	China	Girls and boys	1,472	Obesity/overweight: Chinese Working Group on Obesity	Quartiles of the decimal age of each Tanner stage (>P_25_)	None	8
Xu et al. (29)	China	Girls and boys	2,908	Obesity/overweight: Chinese Working Group on Obesity	Puberty Development Scale (PDS) score >$\bar{x}$ + s	Region, age, whether the only child, family economic status, parents' educational level	9

### Childhood Overweight/Obesity and Early Puberty in Girls

**Figure 2:**
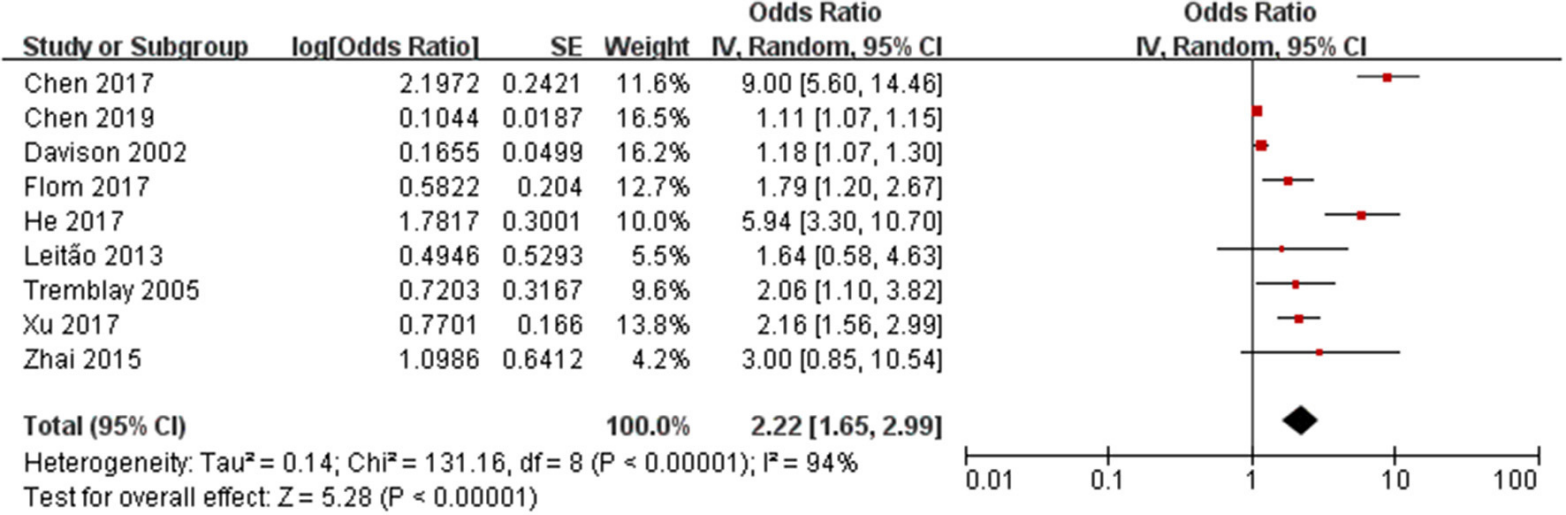
Forest plot of childhood overweight/obesity and the risk of early puberty in girls.

The risk estimates of early puberty in girls associated with childhood overweight/obesity are summarized in Figure 2. A total of 1,104 cases with early puberty in 13,338 girls in childhood were identified. The ORs for the association reported by included studies ranged from 1.11 to 9.00. Meta-analytic pooling of these risk estimates yielded a summary OR of 2.22 (95% CI 1.65–2.99), with substantial heterogeneity (*I*^2^ = 94%, *p* < 0.001). Begg's test did not show a potential publication bias (*z* = 0.42, *p* = 0.677). Sensitivity analysis were performed by repeating the meta-analysis after the exclusion of each included study; results showed that exclusion of any single study did not materially alter the risk estimate of early puberty in girls (ORs ranged between 1.72 and 2.64; see Table 3).

**Table 3:** Sensitivity analysis for the association of childhood overweight/obesity and early puberty in girls.

	**OR**	**95%CI**	**95%CI**	***P*** **-value**	***I***^**2**^ **(%)**
Chen et al. (27)	1.7235	1.3671	2.1728	<0.0001	88.10
Chen et al. (19)	2.6383	1.5142	4.5967	0.0006	93.30
Davison et al. (26)	2.6163	1.4581	4.6944	0.0013	94.70
Flom et al. (20)	2.2988	1.6698	3.1646	<0.0001	94.50
He and Karlberg (7)	1.9609	1.4755	2.6061	<0.0001	93.10
Leitão et al. (31)	2.2658	1.6676	3.0786	<0.0001	94.60
Tremblay and Frigon (30)	2.2428	1.6416	3.0642	<0.0001	94.50
Xu et al. (29)	2.2264	1.6257	3.049	<0.0001	94.00
Zhai et al. (25)	2.1945	1.6218	2.9695	<0.0001	94.60

Subgroup analyses for the association between childhood overweight/obesity and early puberty in girls are shown in Table 4. After subgroup analyses, the variables including study design [test for subgroup differences (TSD): χ^2^= 7.56, *p* = 0.006], sample size (TSD: χ^2^= 37.64, *p* < 0.001), follow-up duration (TSD: χ^2^= 5.21, *p* = 0.02), definition of early puberty (TSD: χ^2^= 49.26, *p* < 0.001), and confounders controlled (TSD: χ^2^= 8.09, *p* = 0.004) were shown to be associated with the between-study heterogeneity. In studies with a case-control design, the risk of early puberty related to childhood overweight/obesity (OR: 4.80, 95% CI: 1.85–12.43; *p*<0.001) was significantly higher than that in studies with a cohort design (OR: 1.24, 95% CI: 1.08–1.43; *p* = 0.020). It should be noted that overweight/obese children were at greater risk of early puberty when the study sample size was greater than 2000 or the follow-up duration was longer than 8 years. When stratified by definition of early puberty, positive associations between childhood overweight/obesity and early puberty were still found in studies defined early puberty by age of breast stage II stage (OR: 6.10, 95% CI: 2.18–17.08), age of each Tanner stage (OR: 5.4, 95% CI: 3.30–10.70), age of menarche (OR: 2.28, 95% CI: 1.03–5.04), PDS score >$\bar{x}$ + s (OR: 2.16, 95% CI: 1.56–2.99), and three criteria in the study (OR: 1.18, 95% CI: 1.07–1.30). In addition, a significantly higher elevation in the risk of early puberty related to childhood overweight/obesity was found in studies with none of the confounders controlled (OR: 4.87, 95% CI: 2.08–11.39; *p* = 0.001) when compared with studies that had one or more confounders controlled (OR: 1.38, 95% CI: 1.15–1.65; *p* < 0.001). Moreover, through subgroup analysis, we found that overweight girls (OR=4.67, 95% CI: 1.60–13.63) enter puberty earlier than obese girls (OR=2.22, 95% CI: 1.65–2.99).

**Table 4:** Subgroup analyses for the association between childhood overweight/obesity and early puberty in girls.

**Subgroup**	**No. of studies**	**OR (95% CI)**	***I***^**2**^ **(%)**	***P*** **-value for heterogeneity**	**Test for subgroup differences**		
					**χ^2^**	***P*** **-value**	***I***^**2**^ **(%)**
**Geographic region**					2.47	0.29	18.9
Europe	1	1.64 [0.58, 4.63]	–	–			
Asia	5	3.23 [1.35, 7.76]	97	<0.001			
North America	3	1.51 [1.04, 2.19]	70	0.04			
**Study design**					7.56	0.006	86.8
Cohort	6	1.24 [1.08, 1.43]	62	0.02			
Case-control	3	4.80 [1.85, 12.43]	92	<0.001			
**Sample size**					37.64	<0.001	97.3
> 2,000	1	9.00[5.60, 14.46]	–	–			
≤ 2,000	8	1.72[1.37, 2.17]	88	<0.001			
**Follow-up duration**					5.21	0.02	80.8
> 8 years	1	1.79[1.20, 2.67]	–	–			
≤ 8 years	5	1.12[1.08,1.16]	49	0.10			
**Age at enrolment**					0.11	0.74	0
≥ 7 years old	4	1.54[0.96,2.45]	55	0.08			
**<** 7 years old	2	1.39[0.93,2.06]	75	0.05			
**Type of exposure**					1.71	0.19	41.4
Obesity	9	2.22 [1.65, 2.99]	94	<0.001			
Overweight	3	4.67 [1.60, 13.63]	81	0.005			
**Definition of overweight/obesity**					0.55	0.76	0
Body mass index	7	2.24 [1.63, 3.06]	95	<0.001			
Skinfold thickness	1	3.00 [0.85, 10.54]	–	–			
Body fat	1	1.64 [0.58, 4.63]	–	–			
**Definition of early puberty**					49.26	<0.001	91.9
Age of menarche	5	2.28 [1.03, 5.04]	95	<0.001			
Age of breast stage II stage	2	6.10 [2.18, 17.08]	61	0.11			
Three criteria in the study^a^	1	1.18 [1.07, 1.30]	–	–			
Age of each Tanner stage	1	5.94 [3.30, 10.70]	–	–			
PDS^b^ score > $\bar{x}$ + s	1	2.16 [1.56, 2.99]	–	–			
**Confounders controlled**					8.09	0.004	87.6
None confounders controlled	3	4.87 [2.08, 11.39]	86	0.001			
One or more confounders controlled	6	1.38 [1.15, 1.65]	80	<0.001			

a * Earlier developers were girls who fulfilled at least two of the following three criteria: (1) highest tertile for estradiol; (2) tanner stage three for breast development; and (3) highest tertile for the puberty development scale (PDS)*.

b *Puberty development scale*.

### Childhood Overweight/Obesity and Early Puberty in Boys

Qualitative analysis identified that boys with high BMI trajectories had a greater risk of early puberty than boys with low BMI trajectories (13). Furthermore, a birth cohort study with 18 years of follow-up found a positive association between obesity and earlier puberty in boys (10) and a cohort and sibling-matched analysis found that higher BMI was associated with earlier age at attaining most pubertal milestones in girls, but only a tendency toward earlier pubertal timing was observed in boys (32).

**Figure 3:**
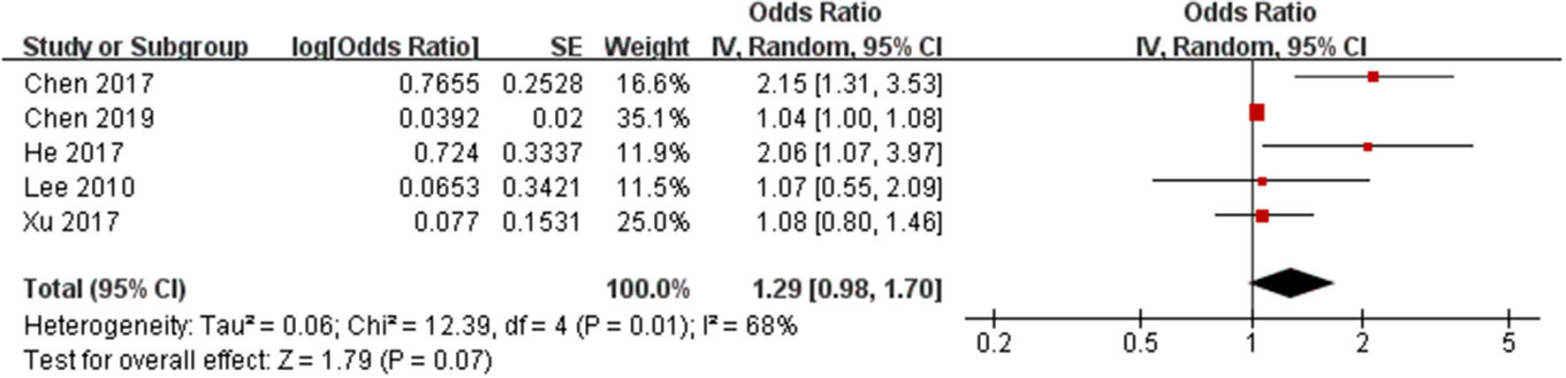
Forest plot of childhood overweight/obesity and the risk of early puberty in boys.

The risk estimates of early puberty in boys associated with childhood overweight/obesity are summarized in Figure 3. A total of 586 cases with early puberty in 12,796 boys in childhood were identified. The ORs for the association reported by included studies ranged from 1.04 to 2.15. Meta-analytic pooling of these risk estimates yielded a summary OR of 1.29 (95% CI 0.98–1.70), with substantial heterogeneity (*I*^2^ = 68%, *p* = 0.01). Begg's test did not show a potential publication bias (*z* = 0.49, *p* = 0.624). Sensitivity analysis were performed by repeating the meta-analysis after the exclusion of each included study; results showed that exclusion of any single study did not materially alter the risk estimate of early puberty in boys (ORs ranged between 1.09 and 1.47; see Table 5).

**Table 5:** Sensitivity analysis for the association of childhood overweight/obesity and early puberty in boys.

	**OR**	**95%CI**	**95%CI**	***P*** **value**	***I***^**2**^ **(%)**
Chen et al. (27)	1.0942	0.9251	1.2942	0.2932	29.50
Chen et al. (19)	1.4665	0.9808	2.1927	0.0621	60.50
He et al. (28)	1.193	0.919	1.5485	0.1849	63.70
Lee et al. (13)	1.3414	0.9738	1.8478	0.0722	75.80
Xu et al. (29)	1.4345	0.9221	2.2317	0.1095	75.70

Subgroup analyses for the association between childhood overweight/obesity and early puberty in boys are shown in Table 6. Due to insufficient data in the included articles, the subgroup analysis of the associate between overweight/obesity and early puberty in boys only including geographic region, study design, sample size, and confounders controlled. After subgroup analyses, the variables including sample size (TSD: χ^2^= 6.40, *p* = 0.01) and confounders controlled (TSD: χ^2^=12.31, *p* < 0.001) were shown to be associated with the between-study heterogeneity. In studies with a sample size greater than 2,000, obese children were at higher risk of entering puberty early (OR: 2.15, 95% CI: 1.31–3.53). When stratified by confounders controlled, positive associations between childhood overweight/obesity and early puberty were still found in studies that have none of the confounders controlled (OR: 2.12, 95% CI: 1.43–3.14), and studies that had one or more confounders controlled (OR: 1.04, 95% CI: 1.00–1.08).

**Table 6:** Subgroup analyses for the association between childhood overweight/obesity and early puberty in boys.

**Subgroup**	**No. of studies**	**OR (95% CI)**	***I***^**2**^ **(%)**	***P*** **-value for heterogeneity**	**Test for subgroup differences**		
					**χ^2^**	***P*** **-value**	***I***^**2**^ **(%)**
**Geographic region**					0.36	0.55	0
Asia	4	1.34 [0.97, 1.85]	76	0.006			
North America	1	1.07 [0.55, 2.09]	–	–			
**Sample size**					6.40	0.01	84.4
> 2,000	1	2.15 [1.31, 3.53]	–	–			
≤ 2,000	4	1.09 [0.93, 1.29]	29	0.24			
**Study design**					2.77	0.1	63.9
Cohort	2	1.04 [1.00, 1.08]	0	0.94			
Case-control	3	1.61 [0.96, 2.68]	72	0.03			
**Confounders controlled**					12.31	<0.001	91.9
None confounders controlled	2	2.12 [1.43, 3.14]	0	0.92			
One or more confounders controlled	3	1.04 [1.00, 1.08]	0	0.97			

## Discussion

In this study, by combining the results of all available data with the conventional method of meta-analysis, we provided evidence that childhood overweight/obesity were associated with a higher risk of early puberty in girls. Specifically, childhood overweight and obesity in girls may increase the risk of early puberty by 4.67-fold, and 2.22-fold, respectively. An increased risk of early puberty related to childhood overweight/obesity was also found in boys, although the estimate was not statistically significant. As far as we know, this study is the most up-to-date and comprehensive meta-analysis evaluating the relationship between childhood overweight/obesity and early puberty in both boys and girls, which can supply helpful information to both parents and primary health care providers, and help to guide relevant policy and advocacy efforts.

To our knowledge, several potential mechanisms have been proposed to explain the relationships between childhood overweight/obesity and early puberty de Ridder et al. suggested obesity in preadolescent children may lead to the improvement of aromatase activity, which can accelerate the conversion of androgens to estrogen (33). Meanwhile, obese children's tissues were exposed to high levels of estrogen, which ultimately leads to early puberty. Previous studies found that obesity may alter hormone secretion and sensitivity, including leptin and insulin. Leptin levels gradually increase with age before puberty, and puberty may be triggered when the threshold is reached (34). Moreover, obese children had higher levels of leptin than lean children, which may have contributed to their earlier puberty (35). In the hypothalamus, leptin can stimulate the secretion of kisspeptin, which in turn activates the hypothalamic-pituitary-gonadal (HPG) axis and promotes the secretion of GnRH from hypothalamic arcuate neurons in a dose-dependent manner, thereby accelerating puberty (36). Based on the underlying mechanisms between the childhood overweight/obesity and early puberty, we ventured to speculate that there might be a link between childhood overweight/obesity and early puberty in boys. Our study is the first time to conduct a meta-analysis of the relationship between childhood overweight/obesity and early puberty in boys, although the association is possible. Future research is needed to confirm the association between childhood overweight/obesity and early puberty in boys based on larger sample size.

A previous meta-analysis based on 69 cases with early puberty in 165 girls who were overweight/obese in childhood detected a significant higher risk of early puberty in relation to childhood overweight/obese with a risk estimate of 2.44 (18) our study validated the relationship on the basis of a larger sample size (13,338 girls and 12,796 boys). Meanwhile, we also analyzed the association between childhood overweight/obese and puberty in boys. Furthermore, through subgroup analysis, we found that individuals who were overweight in childhood (*OR* = 4.67) had a higher risk of early puberty than those who were obesity in childhood (*OR* = 2.22), which is consistent with the results of a cohort and sibling-matched analyses (i.e., the timing of puberty advances for overweight girls and obese girls was 5.5 months and 5.2 months, respectively) (32). The different effect of overweight and obesity on pubertal development in girls leads to speculation that childhood overweight/obesity and pre-puberty may be more than a simple linear correlation, so rising weight is not necessarily accompanied by a constant advance in puberty. Besides, the Frisch hypothesis suggested that menarche occurred at a critical weight, when weight exceeds the threshold, even if weight continues to rise, it's not necessarily accompanied by an increased incidence of early puberty (37). Of note, however, only three studies with a total of 150 cases with early puberty were included in the subgroup analysis for childhood overweight, which may make the analyses imprecise.

The strength of this meta-analysis is the large sample size which involving 13,338 girls and 12,796 boys, and thereby can enhance statistical power, providing more precise and reliable risk estimates. Compared with previous the meta-analysis, our study is more comprehensive since we assessed the association between childhood overweight/obesity and risk of early puberty in both girls and boys. To prevent the omission of relevant literature, a comprehensive and specific search strategy was used at the beginning of this study. Then, a set of more rigorous selection criteria was used in the screening of the selected studies. Besides, detailed analyses were conducted for more diverse subgroups. Finally, more recent studies were contained, which makes our results more reliable to present practices.

On the basis of a large sample size, we received a more convincing result. However, some limitations must be considered. Firstly, only articles published in English were included in our study, which may lead to the omission of articles published in other languages and articles that have not yet been published. Secondly, substantial heterogeneity was found in this meta-analysis. Although subgroup analysis revealed several important sources of heterogeneity, such as study design, sample size, follow-up duration, definition of early puberty and confounders controlled, we still cannot ignore the existence of heterogeneity. Meanwhile, since some subgroup categories contained only one or very few studies, the statistical power is weakened. Thirdly, significant unmeasured confounders might be involved in the observed relation between childhood overweight/obesity and early puberty. Out of the 10 included studies, seven studies have controlled for one or more confounders, such as parental education, family income, and urban or rural. The reproducibility between the confounding factors controlled across these studies was also limited. Considering that some studies included in this meta-analysis have not controlled for the key confounders of early puberty, such as race, birth weight and physical activity, it is possible that the increased risks found in this study partly due to differences in these risk factors of early puberty between overweight/obesity in childhood and the reference groups. Fourth, both case-control and cohort studies were included in our study, case-control studies may appear reverse causality. Case-control studies are widely considered to be prone to recall and selection biases, which restrict the strength and quality of evidence. Compared to studies with a cohort design (OR: 1.24), subgroup analyses showed that the risk of early puberty related to childhood overweight/obesity was significantly higher in case-control studies (OR: 4.80). Fifth, different types of markers of puberty timing and obesity that may have been obtained at a single time-point and/or self-reported, which may lead to measurement errors, for example, the use of BMI as an indirect measure of fat mass in epidemiological studies has its limitations. Children with high muscle mass, such as tall and athletic boys, are incorrectly classified as overweight/obesity based on their BMI, which can lead to false link between overweight/obesity in childhood and early puberty (38). More notably, the children involved in these studies may have already begun puberty, which can also lead to selective bias in the results. Last but not least, there were few original studies on obesity and early puberty in boys, only five articles were included in this meta-analysis, and the definition of puberty in boys is also controversial. Therefore, the results of this study should be extended with caution.

Our finding of an association between childhood overweight/obesity and the risk of early puberty has significant implications for child and adolescent health. In addition, the common view is that early puberty is a complex phenomenon caused by many environmental and genetic factors, as well as interactions among these (39). However, owing to the limitations in the original data, we were unable to analyze genetic factors that contribute to early puberty. Further research should focus not only on the relation of environmental factors and early puberty but also on genetic factors, and gene-environment interactions.

In conclusion, based on the available evidence, our systematic review and meta-analysis showed that for girls the associate between overweight/obesity and early puberty is definite or strong whereas for males, such an association is possible, prompting that future studies need to further explore the possible relationship between overweight/obesity and early puberty in boys.

## Data Availability Statement

The original contributions presented in the study are included in the article/supplementary material, further inquiries can be directed to the corresponding authors.

## Author Contributions

LC and TW: study concept and design and study supervision. XZ, YH, ZY, ZG, SZ, XL, YC, and CY: acquisition, analysis, or interpretation of data. XZ and YH: statistical analysis and drafting of the manuscript. LC, TW, XZ, and YH: critical revision of the manuscript for important intellectual content. LC obtained funding administrative, technical, or material support. All authors contributed to the article and approved the submitted version.

## Funding

This study was supported by National Natural Science Foundation Program of China (Grant Nos. 81973137 and 82173608).

## Conflict of Interest

The authors declare that the research was conducted in the absence of any commercial or financial relationships that could be construed as a potential conflict of interest.

## Publisher's Note

All claims expressed in this article are solely those of the authors and do not necessarily represent those of their affiliated organizations, or those of the publisher, the editors and the reviewers. Any product that may be evaluated in this article, or claim that may be made by its manufacturer, is not guaranteed or endorsed by the publisher.
